# Ametabolic FDG-PET in Splenic Angiosarcoma: A Diagnostic Challenge

**DOI:** 10.5152/tjg.2026.26238

**Published:** 2026-06-05

**Authors:** Burcu Gürbüz, Hilmi Anıl Dinçer, İzzetcan Ulusoy, Muşturay Karçaaltıncaba, Onur Keskin

**Affiliations:** 1 Department of Gastroenterology, Hacettepe University Faculty of Medicine, Ankara, Türkiye; 2 Department of General Surgery, Hacettepe University Faculty of Medicine, Ankara, Türkiye; 3 Department of Pathology, Hacettepe University Faculty of Medicine, Ankara, Türkiye; 4 Department of Radiology, Hacettepe University Faculty of Medicine, Ankara, Türkiye

Dear Editor,

Primary angiosarcoma of the spleen (PAS) is a rare, malignant endothelial neoplasm characterized by aggressive behavior, early metastasis, and poor survival.^1^^,^^2^ Patients may present with abdominal pain, splenomegaly, cytopenias, or hemoperitoneum. Cross-sectional imaging often demonstrates heterogeneous hemorrhagic or necrotic splenic masses that overlap with findings seen in lymphoma, peliosis hepatis, metastatic disease, infectious granulomatous processes, and other vascular tumors. Because metabolic and anatomic imaging findings may be discordant, histopathologic confirmation is often required. In this context, we report a diagnostically challenging case of PAS presenting with hemorrhagic hepatosplenic lesions and ametabolic FDG-PET/CT findings, highlighting that negative metabolic imaging does not exclude malignancy in necrotic vascular tumors. Written informed consent for publication of clinical details and images was obtained.

A 42-year-old woman presented with several weeks of fatigue, progressive jaundice, diffuse abdominal pain, and fever, along with a history of heavy menstrual bleeding. Relevant exposures included a recent tick bite, consumption of unpasteurized milk and cheese, and contact with stagnant water. Approximately 10 days after the tick bite, she developed fever, malaise, and nausea. She had no known chronic systemic disease.

On physical examination, she exhibited icteric sclerae, upper-quadrant abdominal tenderness, bilateral pretibial edema, ecchymoses over the flanks and thighs, and splenomegaly, without peripheral lymphadenopathy. Laboratory evaluation revealed cholestatic liver injury with hyperbilirubinemia (total bilirubin: 8.1 mg/dL), elevated alkaline phosphatase (180 U/L) and gamma-glutamyltransferase (147 U/L) levels, normocytic anemia (hemoglobin: 9.6 g/dL), leukocytosis (15 × 10^9^/L), thrombocytopenia (70 × 10^9^/L), hypoalbuminemia (2.66 g/dL), elevated C-reactive protein (57 mg/L), and mildly prolonged INR (International Normalized Ratio) (1.27). Aspartate aminotransferase was mildly elevated (60 U/L), whereas alanine aminotransferase and tumor markers were within normal limits (20 U/L).

The initial differential diagnosis included zoonotic or vector-borne infections, tuberculosis, hematologic malignancies, metastatic disease, and vascular tumors.

Peripheral blood smear demonstrated mature neutrophilia without blasts or atypical cells, and bone-marrow biopsy findings were interpreted as reactive rather than malignant. Two diagnostic paracenteses yielded hemorrhagic ascites, and cytologic analysis was negative for malignancy. Broad infectious and viral testing, including Crimean-Congo hemorrhagic fever PCR (Polymerase Chain Reaction), TORCH (Toxoplasma, other infections, Rubella, Cytomegalovirus infection, Herpes simplex virus) screening, viral hepatitis serologic testing, and Epstein–Barr virus serologic testing, yielded negative results. Upper endoscopy, echocardiography, and ophthalmologic examination findings were unremarkable.

Ultrasonography demonstrated hepatosplenomegaly with indeterminate hepatic lesions, heterogeneous splenic masses, and ascites (Figure 1A). Contrast-enhanced computed tomography confirmed lobulated splenic lesions with intralesional hemorrhage, capsular and pericapsular fluid collections associated with hemoperitoneum, and multiple hypodense hepatic lesions without biliary dilatation (Figure 1B). Magnetic resonance imaging revealed mixed-signal lesions with blood-product characteristics, fluid–fluid levels, and minimal peripheral enhancement, consistent with a hemorrhagic and necrotic process. FDG-PET/CT demonstrated ametabolic (nonavid) hepatosplenic lesions with no appreciable uptake above background and no additional metabolically active foci (Figure 1C). Percutaneous liver biopsy demonstrated biliary and lobular injury with sinusoidal dilatation and congestion, without granulomas or evidence of malignancy.

Integrating the epidemiology, laboratory profile, and imaging findings, a multidisciplinary meeting was convened involving gastroenterology, infectious diseases, general surgery, and radiology. PAS with hepatic involvement was considered the leading diagnosis. Infectious hepatosplenic disease became less likely given negative microbiologic and viral studies, absence of granulomas, and reactive bone marrow findings. Because the patient’s pain persisted, the splenic tumor burden appeared to progress, and all minimally invasive tests remained nondiagnostic, operative management was pursued.

The resected spleen contained a large, variegated hemorrhagic and necrotic mass. Histopathologic examination demonstrated a malignant vasoformative neoplasm characterized by marked cytologic atypia, pleomorphism, irregular anastomosing vascular channels, and extensive necrosis. Immunohistochemistry staining showed strong positivity for CD31 and ERG (Erythroblast Transformation-specific related Gene) , supporting the diagnosis of PAS over epithelioid hemangioendothelioma (EHE). Additional molecular testing for EHE-associated alterations, including WWTR1-CAMTA1 fusion, was not performed. The patient was subsequently referred to medical oncology, and systemic chemotherapy was planned to address presumed hepatic involvement (Figure 2).

In this case, the combination of multifocal hemorrhagic splenic masses, hemoperitoneum, and synchronous hepatic lesions is highly suggestive of PAS, although relevant mimics include peliosis hepatis, lymphoma, metastatic carcinoma, infectious hepatosplenic disease, and other vascular tumors.^1^^-^^3^

Peliosis hepatis may manifest as blood-filled spaces in the liver and, rarely, the spleen; however, it lacks endothelial atypia and destructive growth patterns. Lymphoma and metastatic carcinoma were considered but were inconsistent with the malignant vasoformative histology and endothelial immunophenotype. Infectious etiologies (e.g., tuberculosis, hydatid disease, and pyogenic abscess) were not supported by serologic, microbiologic, or clinical evolution. Benign and intermediate vascular lesions typically demonstrate bland cytology and distinct architectural patterns.

EHE should also be considered in the pathologic differential diagnosis because both EHE and angiosarcoma can express CD31 and ERG. In this case, marked cytologic atypia, pleomorphism, destructive irregular vasoformative architecture, and extensive necrosis favored a diagnosis of PAS over EHE; molecular testing for WWTR1-CAMTA1 fusion was not performed.

Although FDG-PET/CT is frequently incorporated into oncologic staging, data regarding uptake characteristics in larger series of PAS remain limited. Published case series emphasize imaging heterogeneity, but the proportion of PET-negative lesions has not been consistently reported, likely owing to disease rarity and variable proportions of viable tumor and necrosis. False-negative FDG-PET findings have been described in angiosarcoma, supporting the possibility of low uptake in necrosis-rich lesions.^4^

A key educational feature of this case is the false-negative metabolic imaging pattern. In this patient, extensive gross and microscopic necrosis provided a biologic explanation for the ametabolic PET findings.

A radiologic–pathologic review has proposed an algorithmic approach to splenic lesions;^3^ however, its applicability in the present case was limited because the lesion demonstrated overlapping hemorrhagic and necrotic features across ultrasonography, CT, and MRI, along with ametabolic PET findings, rendering imaging alone insufficient for definitive classification.

Published literature demonstrates substantial heterogeneity in the imaging characteristics of splenic angiosarcoma. Furthermore, recent reports have shown that benign hepatic mass lesions may mimic metastatic or other malignant processes, further emphasizing the importance of histopathologic confirmation in atypical hepatobiliary presentations.^5^ Although a radiologic–pathologic review has proposed an algorithmic approach to splenic lesions, its applicability in the present case was limited because overlapping hemorrhagic and necrotic features across ultrasonography, CT, and MRI, together with ametabolic PET findings, rendered imaging alone insufficient for definitive classification.^3^

This case further highlights the value of multidisciplinary evaluation in resolving complex diagnostic pathways involving discordant imaging, broad infectious differentials, and nondiagnostic preliminary investigations.^6^ When metabolic and anatomic imaging conflict, histopathologic confirmation remains definitive, and splenectomy may be both diagnostic and therapeutic.

## Figures and Tables

**Figure 1.**
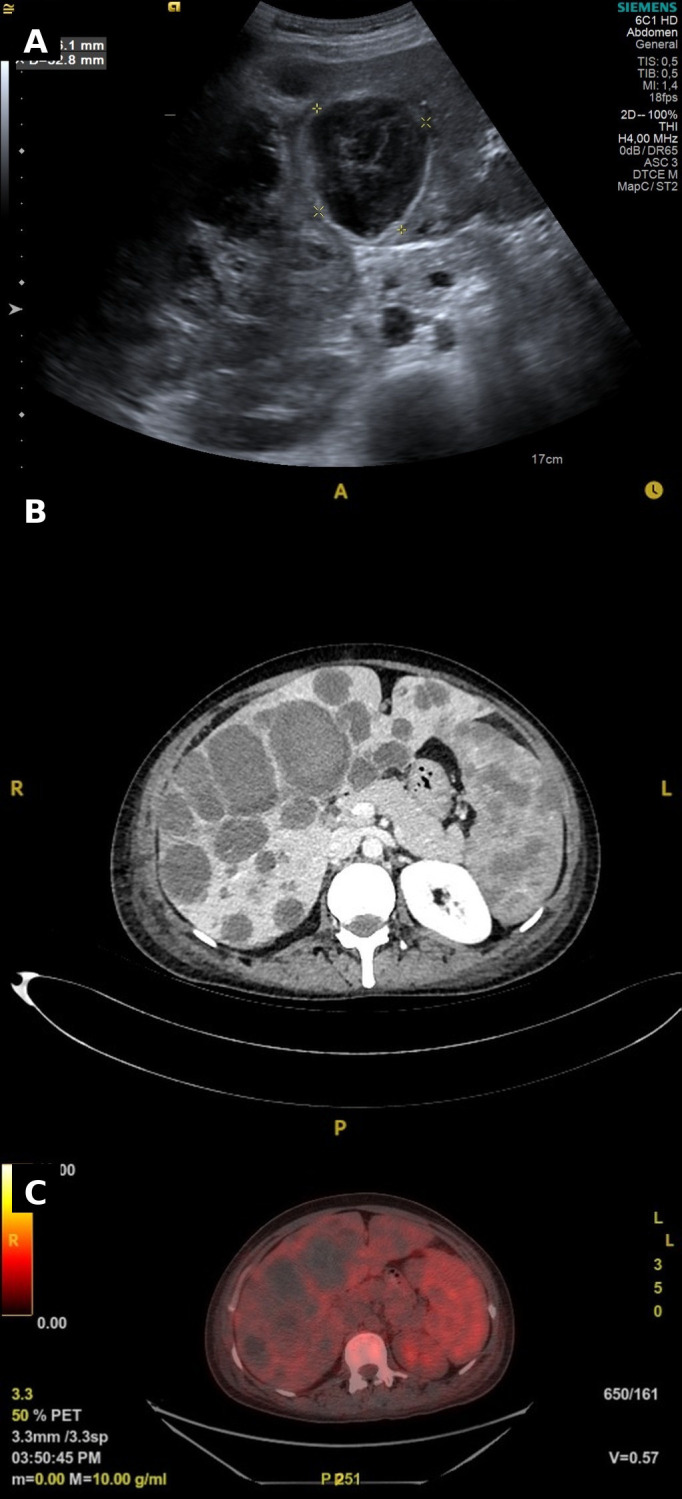
Imaging. (A) Ultrasound demonstrates hypoechoic hepatic lesions, heterogeneous hypoechoic splenic masses with internal echoes, and free fluid. (B) Axial and coronal contrast-enhanced CT images demonstrate lobulated, heterogeneous splenic lesions with high-attenuation foci (intralesional hemorrhage) and hemoperitoneum. (C) Ametabolic appearance of the splenic and hepatic lesions on FDEG-PET/CT, illustrating a potential false-negative PET result in necrotic vascular tumors. CT, computed tomography; PET, positron emission tomography.

**Figure 2.**
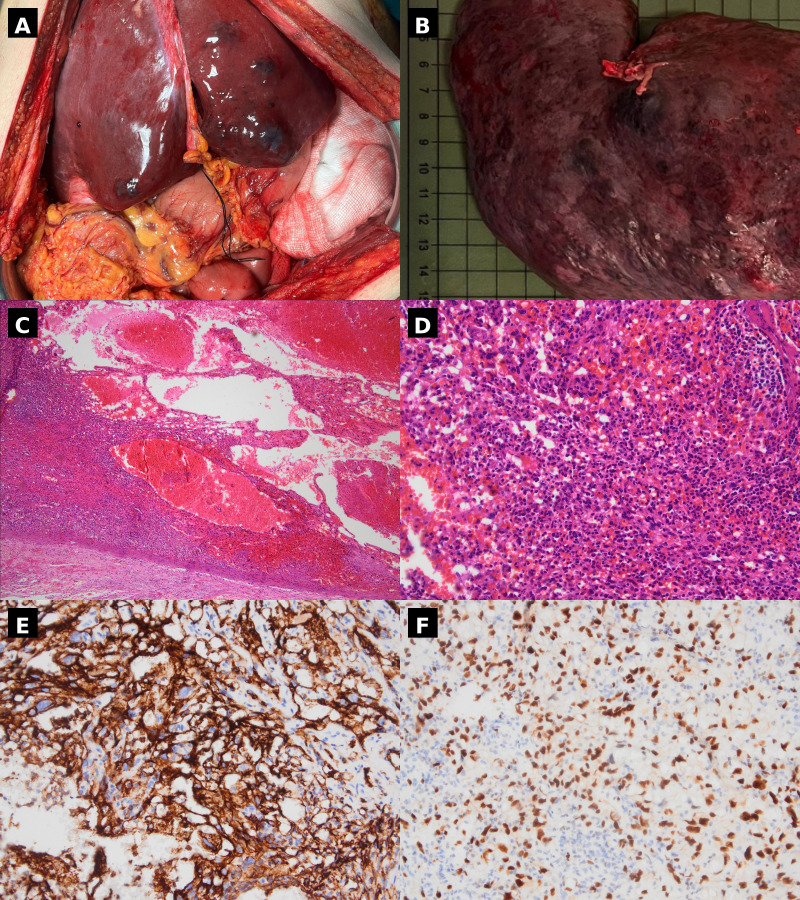
Splenectomy and pathology. (A) Intraoperative appearance of the liver and metastatic lesions. (B) Gross splenectomy specimen demonstrating a markedly enlarged spleen with an irregular hemorrhagic mass and a disrupted capsular surface. (C) Low-power hematoxylin and eosin (H&E)-stained section demonstrating extensive hemorrhage, blood-filled spaces, and areas of necrosis within the tumor. (D) Higher-power H&E-stained image showing a malignant vasoformative proliferation composed of atypical, pleomorphic endothelial cells forming irregular, anastomosing vascular channels. (E) CD31 immunohistochemistry demonstrating diffuse, strong membranous positivity in neoplastic endothelial cells. (F) ERG immunohistochemistry demonstrating diffuse nuclear positivity, confirming endothelial differentiation.

## Data Availability

The data that support the findings of this study are available on request from the corresponding author.
